# Appraising systematic reviews: a comprehensive guide to ensuring validity and reliability

**DOI:** 10.3389/frma.2023.1268045

**Published:** 2023-12-21

**Authors:** Nour Shaheen, Ahmed Shaheen, Alaa Ramadan, Mahmoud Tarek Hefnawy, Abdelraouf Ramadan, Ismail A. Ibrahim, Maged Elsayed Hassanein, Mohamed E. Ashour, Oliver Flouty

**Affiliations:** ^1^Alexandria Faculty of Medicine, Alexandria University, Alexandria, Egypt; ^2^Faculty of Medicine, South Valley University, Qena, Egypt; ^3^Faculty of Medicine, Zagazig University, Zagazig, Egypt; ^4^Medical Research Group of Egypt, Cairo, Egypt; ^5^Faculty of Medicine, Cairo University, Cairo, Egypt; ^6^Faculty of Health Sciences, Fenerbahce University, Istanbul, Türkiye; ^7^Department of Neurosurgery and Brain Repair, University of South Florida, Tampa, FL, United States

**Keywords:** systematic review, quality assessment, systematic review methodology, systematic review appraisal, bias evaluation

## Abstract

Systematic reviews play a crucial role in evidence-based practices as they consolidate research findings to inform decision-making. However, it is essential to assess the quality of systematic reviews to prevent biased or inaccurate conclusions. This paper underscores the importance of adhering to recognized guidelines, such as the PRISMA statement and Cochrane Handbook. These recommendations advocate for systematic approaches and emphasize the documentation of critical components, including the search strategy and study selection. A thorough evaluation of methodologies, research quality, and overall evidence strength is essential during the appraisal process. Identifying potential sources of bias and review limitations, such as selective reporting or trial heterogeneity, is facilitated by tools like the Cochrane Risk of Bias and the AMSTAR 2 checklist. The assessment of included studies emphasizes formulating clear research questions and employing appropriate search strategies to construct robust reviews. Relevance and bias reduction are ensured through meticulous selection of inclusion and exclusion criteria. Accurate data synthesis, including appropriate data extraction and analysis, is necessary for drawing reliable conclusions. Meta-analysis, a statistical method for aggregating trial findings, improves the precision of treatment impact estimates. Systematic reviews should consider crucial factors such as addressing biases, disclosing conflicts of interest, and acknowledging review and methodological limitations. This paper aims to enhance the reliability of systematic reviews, ultimately improving decision-making in healthcare, public policy, and other domains. It provides academics, practitioners, and policymakers with a comprehensive understanding of the evaluation process, empowering them to make well-informed decisions based on robust data.

## Introduction

A systematic review is a comprehensive and rigorous approach to identifying, selecting, and analyzing relevant literature on a specific research question or topic (Muka et al., 2020). In the field of evidence-based practice, systematic reviews are an essential tool for synthesizing and analyzing research findings. The process involves searching multiple databases, screening articles for eligibility, assessing the quality of included studies, and synthesizing the results. This approach minimizes the risk of bias and increases the validity and reliability of the review findings. To ensure that a review is systematic, it is critical to adhere to established guidelines such as the Cochrane Handbook for Systematic Reviews of Interventions or the Preferred Reporting Items for Systematic Reviews and Meta-Analyses (PRISMA) statement. These guidelines provide a framework for performing and creating systematic reviews, as well as ensuring that important components of the review process, such as search strategy and study selection, are well documented. Systematic reviews are increasingly being used to inform decision-making in healthcare, public policy, and other fields (Cumpston et al., 2019; Muka et al., 2020). As such, it is crucial to appraise the quality of systematic reviews to ensure that their findings are valid and reliable (Muka et al., 2020). Appraising systematic reviews involves critically evaluating the methods used in the review process, the quality of the included studies, and the overall strength of the evidence presented (Linares-Espinós et al., 2018). Once a systematic review is completed, A meta-analysis can be used for summarizing and analyzing the data. A meta-analysis is a statistical approach for combining the findings of many studies to determine the overall effect size. It is an essential technique for synthesizing evidence from several research and can provide more precise treatment impact estimates than individual studies.

The importance of appraising systematic reviews cannot be overstated. Flawed or biased systematic reviews can lead to incorrect conclusions and misguided decision-making. Appraising systematic reviews helps to identify potential sources of bias or limitations in the review process and can help to guide future research in the field (Page et al., 2021).

Appraising systematic reviews involves several steps that evaluate the review's quality as well as the amount to which the review authors adhered to established guidelines for conducting a systematic review. Identifying the research topic and inclusion criteria, looking for relevant papers, analyzing the quality of the included studies, synthesizing the findings of the studies, and evaluating the overall quality of the review are all processes in reviewing a systematic review.

The assessment process can be facilitated by several tools and checklists, including the Cochrane Risk of Bias tool and the AMSTAR 2 checklist. These methods may help in locating potential bias-inducing factors in the review, such as selective reporting of results or the inability to consider study heterogeneity (Shea et al., 2017). This paper aims to provide a comprehensive overview of the process of appraising systematic reviews. It covers the key elements of systematic reviews, explores biases that can impact them, and presents tools to assess these biases. The paper aims to improve the quality and reliability of systematic reviews, ultimately enhancing decision-making in various fields.

## Quality assessment of studies included in the systematic reviews

### Clearly defined research question

Choosing a research question is the first cornerstone for building an interesting research project, it helps readers decide whether they should read the text or not. Defining a research question constitutes a future outlook for all the next steps involved in the existence of evidence. To define a strong research question, we may examine the literature to find knowledge gaps or if the additional analysis would fill in gaps and provide value. To achieve this goal, we begin with reviewing the literature which entails analyzing the literature on a specific subject only if it supports the claim that a new study will add anything novel or significant to the evidence of knowledge (Siddaway et al., 2019). We can use some tools that can help define and analyze the study question which is shown in Table 1.

**Table 1:** Tools for defining the research question.

**Type of research**	**Used tool**	**Description**
Evidence-based clinical practice; Schardt et al. (2007)	PICO (s)	P Patient problem I Intervention C Comparison O Outcome (s)
Qualitative research questions; O'Connor et al. (2014)	PEO	P Population E Exposure O Outcome
Mixed-methods research; Cooke et al. (2012)	SPIDER	S Sample PI Phenomenon of interest D Design E Evaluation R Research type

### Adequate and relevant search strategy

The systematic review should be anchored by a meticulously designed search strategy, which will be thoroughly elucidated in the methods section of this paper. The search method is instrumental in retrieving the bulk of research that will undergo evaluation for eligibility and inclusion. Even in cases where a systematic review already exists and does not require updating, this initial search process serves multiple purposes it familiarizes researchers with the existing literature, saves valuable time by avoiding redundant efforts, and aids in determining the necessity for an updated systematic review (Bramer et al., 2018).

How to conduct an adequate search strategy (Skinner, 2023)?

**Figure 1:**
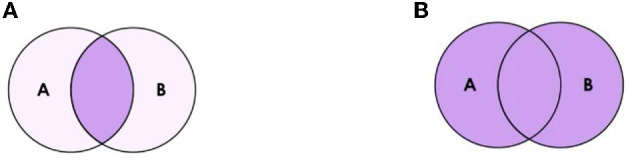
Boolean operators; **(A)** describe using AND in the search strategy, **(B)** describe using OR.

1. Identify the research question.
2. Identify the key concepts and search terms.
3. Phrase searching, Boolean operators as shown in Figure 1.
4. Pilot search strategy and monitor its development.
5. Adapt search syntax for different databases.

### Appropriate inclusion and exclusion criteria

The inclusion and exclusion criteria depend on the topic of the systematic review by targeting a certain population that may have a common range of age, gender, follow-up duration or characteristics involved like a disease or drug administration. Before even reading the literature, they are unaffected by existing research. While planning a study, it is crucial that researchers not only choose the right inclusion and exclusion criteria but also consider how those choices would affect the external validity of the study's findings (Patino and Ferreira, 2018).

To lessen this possible source of bias, efforts are occasionally made to find and include non-English-language literature in systematic reviews. Nonetheless, there is some evidence that, at least in the field of conventional medicine, only incorporating English-language papers does not bias meta-analyses (Skinner, 2023).

A flow diagram outlining the process of finding and sorting through relevant literature is a best practice for systematic reviews (e.g., a PRISMA flow diagram) (Liberati et al., 2009). This should give a brief description of the number of studies included and excluded at each stage of the process.

Embase, SCOPUS, Web of Science, and Cochrane Central should be included in at least four online databases when conducting a literature search. Every database has a unique format for expressing a search method. Selecting the right databases is essential to carrying out an exhaustive literature search because it gives researchers access to a wider variety of sources. These databases cover a distinct collection of journals, conference proceedings, and other scholarly publications, and each one specializes in a different field. For example, PubMed focuses on biomedical literature, while IEEE Xplore specializes in engineering and technology-related publications. By utilizing multiple databases, researchers can ensure a comprehensive search that encompasses diverse perspectives and disciplines relevant to their research topic (Gusenbauer, 2022).

Additionally, every database expresses a search strategy in a unique way. This implies that different databases may have different search operators, search syntax, and query construction techniques. For instance, while some databases use Boolean operators to combine search terms, others may require the use of specific symbols or syntax. It is essential for researchers to familiarize themselves with the search functionalities and specificities of each database they utilize. By understanding and adapting to these various formats, researchers can optimize their search queries and effectively retrieve the required data from each database (Gusenbauer and Haddaway, 2020).

To ensure the robustness of the literature search, it is crucial for the authors to select a database that is comprehensive, up-to-date, and aligned with the scope of their review, including the desired document types. A comprehensive database selection should include both subject-specific and multidisciplinary databases to minimize the risk of missing relevant studies. For example, if the research topic falls within the field of psychology, databases such as PsycINFO and Scopus can provide valuable insights from psychology-specific journals as well as interdisciplinary publications. Furthermore, prioritizing databases that are regularly updated enables access to the most recent literature, particularly in rapidly evolving fields. This is important to ensure that the review incorporates the latest findings and developments, enhancing the relevance and currency of the research. By considering these factors and making informed decisions about database selection, researchers can conduct a comprehensive literature search that encompasses diverse sources, incorporates up-to-date information, and aligns with the scope and objectives of their review (Rethlefsen et al., 2021; Visser et al., 2021).

When it comes to resources for evidence synthesis, the Cochrane Handbook is considered the gold standard. It provides advice on all aspects of the systematic review procedure, including developing research questions, creating search plans, determining bias risk, collecting and evaluating data, and interpreting results. To ensure the greatest caliber of systematic reviews, the handbook offers vital information on standards, procedures, and best practices (Cumpston et al., 2019).

### Quality assessment of studies included

For doctors, researchers, and policymakers, evaluating the quality of research is crucial. Study quality assessment has been used to; establish a minimum quality threshold for the selection of primary studies for systematic reviews, investigate quality differences in study results, weigh study results proportionate to study quality in meta-analyses, direct interpretation of findings, help assess the strength of inferences; and direct recommendations for future research and clinical practice (Chien and Norman, 2009).

Various methodological quality assessment tools are used for various research design types. During the study process, biases such as selection bias, performance bias, detection bias, attrition bias, reporting bias, and others may have an impact on the internal validity (Cochrane Handbook for Systematic Reviews of Interventions, 2023).

Bias and erroneous results may be introduced by primary studies that were included in the evaluation but had poor methodology and reporting quality. Hence, a reliable evaluation of study quality by two impartial reviewers is necessary to ensure accuracy. To minimize bias as a reviewer, it's important to carefully assess the risk of all types of bias such as Publication, Selection, Performance, Detection and Reporting bias in each included study, as well as in the overall review process. This can involve using the validated tools that are mentioned in Table 2 to assess the risk of bias in the overall review. Additionally, it's important to critically evaluate the search strategy, study selection process, and data analysis methods used in the review to ensure that they are comprehensive, unbiased, and well-documented. Quality Assessment Tools for Systematic Reviews are shown in Table 2.

**Table 2:** Quality assessment tools for systematic reviews.

**Tool**	**Assessing the risk of bias**	**According to**
Rob 2; Cochrane Bias (2023)	The quality of RCTs included in systematic reviews and risk of bias.	Five domains, including randomization process, deviations from intended interventions, missing outcome data, measurement of outcomes, and selection of reported results.
NOS; Lo et al. (2014)	The quality of observational studies and to guide the interpretation and application of study findings in clinical practice and policy.	Three domains, including the selection of the study groups, the comparability of the groups, and the ascertainment of either the exposure or outcome of interest
AHRQ or SRDR; Leavy et al. (2019)	To support the conduct of Effectiveness and Comparative Effectiveness Reviews (CERs).	Steps involved in the creation of a systematic review.
GRADE; Drukker et al. (2021), Guyatt et al. (2008)	The quality of evidence and strength of recommendations in healthcare.	The approach categorizes the certainty of evidence as high, moderate, low, or very low, and the strength of recommendations as strong or weak.
OCEBM; Drukker et al. (2021)	They were designed to help clinicians, researchers, and patients quickly and efficiently appraise the quality of evidence without resorting to pre-appraised sources	The OCEBM Levels of Evidence cover the entire range of clinical questions, from level 1 (the highest) to level 5 (the lowest)
OSQE; Němcová and Němcová (2013)	The OSQE also has a scoring system that uses stars and veto cells to indicate the quality of each item.	The OSQE has three versions cohort, case–control, and cross-sectional. Each version includes items that are relevant to that type of study design, such as the representativeness of the study population, the validity of the independent and dependent variables, and the statistical methods used
OCEBM (2023)	The Oxford Center for Evidence-Based Medicine	The Levels of Evidence (levels I-V)are based on a hierarchy of study designs, from the most reliable (Level 1) to the least reliable (Level 5), and cover questions about prevalence, diagnosis, prognosis, treatment, harms, and screening.

### Synthesis of data conducted appropriately (data extraction)

The type of data being handled determines how the data is gathered from the included studies, synthesized, and displayed. If you have quantitative information, the common tools used to summarize data include presenting the results in tables, and charts such as pie-charts or forest plots. If you have qualitative information, the common tools used to summarize data include textual descriptions such as written words or content analysis. The goal of a meta-analysis is to produce an overall summary effect of the data by methodically evaluating and merging the findings of two or more related investigations. Only a small portion of the systematic review process involves the statistical combining of data using meta-analyses, and you should consider if it is suitable before running a meta-analysis on individual results from different research. Depending on how many outcomes you've identified to address the review topic, a systematic review may include several meta-analyses. The benefits of meta-analysis could be a tool to improve statistical power compared to less complex techniques, increase accuracy, look into the causes of differences between studies, and weigh study data by how much and how significantly they add to the analysis (Munn et al., 2014).

### Risk of bias in the systematic review

#### Risk of bias in systematic review

The Risk of Bias in Systematic Reviews (ROBIS) tool is a widely used method to assess the risk of bias in systematic reviews. Both the risk of bias in the review processes and the suitability of the review to the user's research topic are assessed by ROBIS. It evaluates the degree to which review methods lessen the likelihood of bias in the summary estimates and review conclusions as well as the extent to which the research questions the review addressed are relevant to the user's research topic. Inconsistencies in the preparation, execution, or analysis of the review can introduce bias and provide unbalanced results. The review's evidence might not be as pertinent if it differs from the user's research query. To guarantee that the systematic review's findings are reliable and pertinent to the current research issue, it is crucial to assess the systematic review's risk of bias and applicability using a standardized tool like ROBIS. The tool is finished in three stages:

(a) Establish relevancy (optional).
(b) Point out issues with the review process via:

Domain 1 requirements for study eligibility describe the study's eligibility requirements, including any eligibility limitations, and whether there was evidence that the study's goals and eligibility requirements were met.

Domain 2 study identification and selection describe the procedures for selecting and identifying studies (e.g., number of reviewers involved).

Domain 3 study evaluation and data gathering describe the methods used to collect the data, the data that was taken from studies or gathered in other ways, how the risk of bias was evaluated (such as the number of reviewers participating), and the instrument that was used to do so.

Domain 4 Analysis and Conclusions Describe synthesis methods.

(c) assess the review's potential for bias Summarize the concerns identified during the Phase 2 assessment.

#### Risk of bias 2 (ROB)

This is the suggested tool for evaluating the quality and potential for bias in systematic reviews that have been submitted to Cochrane. ROB-2 is the suggested instrument for assessing the possibility of bias in randomized trials included in Cochrane Reviews. ROB-2 is organized into a predetermined set of bias domains with a specific focus on various aspects of trial design, conduct, and reporting. A series of inquiries (referred to as “signaling questions”) within each domain seeks to elicit details regarding trial characteristics that are important to the risk of bias. The RoB-2 assessment of bias is particular to a single trial outcome (and is therefore outcome-based). This sets it apart from the original risk of bias tool, along with other crucial distinctions. For these reasons, certain crucial factors must be defined in advance in the protocol; otherwise, you run the danger of utilizing the tool improperly.

Its Aspects:

- Randomization process.
- Deviations from intended interventions.
- Missing outcome data.
- Measurement of the outcome.
- Selection of the reported result.

### Conflicts of interest disclosed

Disclosure is widely used as a way to manage a competing interest, which is a significant cause of bias in research. Taking into account the significance of systematic reviews and the differing incidence of conflicting interests in various research disciplines. For researchers, reviewers, and editors, the recognition and declaration of competing interests, particularly nonfinancial interests, continues to be difficult. To identify and reduce potential conflicting interests in systematic reviews, the International Council of Medical Journal Editors (ICMJE) must continue to create more effective and efficient tools (Yu et al., 2020).

### Limitations acknowledge

Study limitations represent flaws in a research design that could have an impact on the findings and conclusions of the study. Researchers have a responsibility to the academic community to fully and honestly disclose any limitations of a study that they provide. They should prompt the reader to consider opportunities for future improvements. The presentation of limitations should outline any potential restrictions, clarify their implications, list feasible substitute strategies, and outline actions made to lessen the restrictions. Too frequently, writers leave out these other crucial components in favor of merely listing potential restrictions (Ross and Zaidi, 2019).

#### Possible methodological limitations

##### Sample size

The determination of sample size in a study is contingent upon the nature of the research problem under investigation. It is important to note that if the sample size is insufficient, it may impede the identification of significant relationships within the data. Statistical tests typically require a larger sample size to ensure a representative distribution of the population and to enable the generalization or transferability of results to specific groups. However, it is worth mentioning that the relevance of sample size in qualitative research is generally diminished and should be discussed within the context of the research problem.

##### Lack of available and/or reliable data

In situations where data is lacking or deemed unreliable, it may necessitate constraining the analysis scope and sample size. The absence of adequate data can present a significant challenge in discerning meaningful trends and establishing meaningful relationships. It is imperative to not only describe these limitations but also provide a sound rationale for the absence or unreliability of the data. Rather than succumbing to frustration, it is important to view these limitations as opportunities to identify the need for future research and propose alternative data-gathering methodologies.

##### Lack of prior research studies on the topic

The inclusion of prior research studies forms the foundation for the literature review and facilitates an understanding of the research problem under investigation. Depending on the recency or breadth of the research topic, there may be limited or no existing research available. However, it is recommended to consult with a librarian before assuming the absence of prior research. In cases where a librarian confirms the scarcity of existing research, it may be necessary to develop a new research typology, such as employing an exploratory research design. It should be emphasized that identifying this limitation presents an important opportunity to identify gaps in the literature and articulate the need for further research.

##### Data collection measures

Occasionally, during the interpretation of findings, it may become apparent that the data collection method used hindered a comprehensive analysis of the results. For instance, there might be regret over the omission of a specific question in a survey that, in hindsight, could have addressed a pertinent issue that emerged later in the study. It is crucial to acknowledge such deficiencies and express the necessity for future researchers to revise the data-gathering methods accordingly.

##### Self-reported data

Whether relying on pre-existing data or conducting qualitative research with data collection, self-reported data is limited by the inability to independently verify it. In other words, the accuracy of what individuals express, be it through interviews, focus groups, or questionnaires, is accepted at face value. However, self-reported data is susceptible to several potential sources of bias that should be recognized and highlighted as limitations. These biases include selective memory (remembering or forgetting experiences or events from the past), telescoping (recalling events at a different time than they occurred), attribution (attributing positive events to personal agency but negative events to external forces), and exaggeration (representing outcomes or events as more significant than suggested by other data). These biases become evident when they diverge from data obtained from other sources.

### Possible limitations of the researcher

#### Small sample size

The selection of the sample size in a study is contingent upon the specific research subject being investigated. It is important to acknowledge that identifying significant associations within the data can be challenging when dealing with a small sample size.

#### Lack of readily accessible data

If there is a dearth of reliable data, it may be necessary to limit the scope of the investigation.

#### Absence of previous research studies on the subject

The absence of prior research studies hampers the foundation of the literature evaluation and the overall understanding of the research challenge.

#### Data collection method employed

The choice of data collection method utilized in the study is a factor that should be considered (Botes, 2002).

## Quality assessment of the systematic review

### Critical Appraisal Skills Programme

The Critical Appraisal Skills Programme (CASP) is a widely used tool developed in the UK to evaluate the quality and validity of research studies. It provides a structured way for evaluating various study designs, with particular inquiries and standards to gauge methodological rigor. CASP encourages evidence-based practice and critical thinking in a variety of sectors by assisting researchers and healthcare practitioners in making judgements about the validity and relevance of study findings (CASP, 2023). CASP's 10 items as in Table 1 (see Supplementary File 1). The Supplementary File includes the original checklist of CASP.

There are 10 items of CASP as follows:

Item 1 Did the review address a clearly focused question?
Item 2 Did the authors search for the appropriate papers?
Item 3 Do you think all the important, relevant studies were included?
Item 4 Did the authors of the review assess the quality of the research they incorporated in sufficient detail?
Item 5 Was it acceptable to combine the review's findings, if such is what happened?
Item 6 What are the review's general findings?
Item 7 How precise are the findings?
Item 8 Can the results be applied to the local population?
Item 9 Were all potential outcomes considered?
Item 10 Do the costs and harms outweigh the benefits?

Rating the level of overall satisfaction with the review's findings:

High: no, or just one non-critical flaw the systematic review accurately and thoroughly summarizes the findings of all available studies and deals with the relevant subject.

Moderate: the systematic review has several non-critical flaws^*^, but none are significant. It might give a reliable summary of the outcomes of the available studies that were considered for the evaluation.

Low: the review contains one critical flaw and may not provide an accurate and thorough evaluation of the research that is currently available and addresses the relevant subject.

Critically Low With or without non-critical problems, the review has more than one critical flaw and cannot be relied upon to give an accurate and thorough account of the available research.

Applying the Critical Appraisal Skills Programme (CAPS) methodology or strategy is the next stage. The well-known CAPS programme offers instruction and materials to help people build critical assessment abilities for assessing the quality of research findings (CASP, 2023).

### Assessing the methodological quality of systematic reviews (AMSTAR 2)

AMSTAR is a well-known tool for critically evaluating ONLY systematic reviews of randomized controlled clinical trials and is used to measure the effectiveness of interventions. Assessing the Methodological Quality of Systematic Reviews (AMSTAR 2), which was developed further to enable the evaluation of systematic reviews of randomized and non-randomized studies of healthcare interventions, is frequently used to critically evaluate systematic reviews.

Rapid publication growth in systematic reviews of studies of healthcare interventions has led to a significant increase in their use in clinical and policy decisions. Systematic reviews are increasingly including nonrandomized studies of interventions and are subject to a wide range of biases. It's critical that users can tell when a review is of high caliber. There are numerous instruments that have been created to assess various components of reviews, but there aren't many that offer a thorough critical critique. AMSTAR was developed as a tool for evaluating systematic reviews of randomized trials. AMSTAR's 16 items (see Supplementary File 2). The Supplementary File includes the original checklist of AMSTAR.

There are 16 items if AMSTAR 2:

Item 1 were the PICO components included in the research questions and inclusion standards for the review?
Item 2 did the review report explicitly mention that the review procedures were created before the review was conducted, and did the report provide justification for any significant deviations from the protocol?
Item 3 did the review authors explain their selection of the study designs for inclusion in the review?
Item 4 did the review authors use a comprehensive literature search strategy?
Item 5 did the review authors perform study selection in duplicate?
Item 6 did the review authors perform data extraction in duplicate?
Item 7 did the review authors list the studies they excluded and explain their decisions?
Item 8 did the review writers adequately characterize the included studies?
Item 9 did the review authors' method for determining the risk of bias (RoB) in each study they included in the review meet your standards?
Item 10 did the review authors disclose the funding sources for the studies they reviewed?
Item 11 if a meta-analysis was justified, did the review writers combine the results statistically using the right methods?
Item 12 if a meta-analysis was conducted, did the review authors consider how the outcomes of individual studies' risk of bias (RoB) would affect the findings of the meta-analysis or other evidence synthesis?
Item 13 when interpreting or presenting the review's findings, did the authors take into account RoB in individual studies?
Item 14 did the review authors explain and clarify any apparent heterogeneity in the review's results satisfactorily?
Item 15 if they used quantitative synthesis, did the review authors sufficiently examine publication bias (small study bias) and indicate how it can affect the review's findings?
Item 16 did the review authors disclose any potential conflicts of interest, including any funds they may have received for the review's execution?

Critical domains for AMSTAR 2:

Before the review's start, the protocol was registered (item 2).
The effectiveness of the literature search (item 4).
The rationale behind excluding particular studies (item 7).
Potential for bias in specific studies that are part of the review (item 9).
Suitability of meta-analytical techniques (item 11).
Taking into account the possibility of bias when assessing the review's findings (item 13).
Evaluation of the presence and potential effects of publication bias (item 15).
  The summary of critical appraisal tools and methodologies for evidence-based research is shown in Table 3.

**Table 3:** Summary of critical appraisal tools and methodologies for evidence-based research.

• The Center for Evidence-Based Medicine (CEBM): a collection of critical appraisal tools for all types of studies, as well as examples of their application. • Grading of recommendations, assessments, development, and evaluations GRADE: To assess the level of evidence used in medical research and decision-making. • The Joanna Briggs Institute (JBI) Critical Tools: to assess the trustworthiness, relevance, and results of published papers. • The Newcastle-Ottawa Scale (NOS): to assess the quality of non-randomized studies in meta-analyses. • The Quality Assessment of Diagnostic Accuracy Studies-2 (QUADAS-2): This tool examines studies of diagnostic acuity in four areas the index test, the reference standard, patient selection, and flow and timing. • Strengthening the Reporting of Observational Studies in Epidemiology (STROBE): to address cohort, case-control, and cross-sectional studies. • The Systematic Review Center for Laboratory Animal Experimentation (SYRCLE): to improve the ethical and scientific quality of laboratory animal experimentation through rigorous systematic reviews.

### Challenges in appraising systematic reviews

#### Heterogeneity

Heterogeneity is a critical consideration in systematic reviews and meta-analysis, referring to the variation or diversity of studies included in a research paper (Analysis of heterogeneity in a systematic review using meta-regression technique—PubMed). The presence of heterogeneity must be carefully evaluated, as a high degree of heterogeneity may indicate limitations in conducting a systematic review and meta-analysis. Variations in included studies can be categorized into clinical variations, methodological variations, and statistical variations. Clinical variations encompass differences in participants, outcomes, or interventions. Methodological variations pertain to variations in study designs, measurement tools, or risk of bias. Statistical variations refer to differences in the evaluation of outcomes across studies. It is important to address heterogeneity adequately to prevent misleading results, such as underestimation or overestimation of findings (Analysis of heterogeneity in a systematic review using meta-regression technique—PubMed). Three methods commonly used to assess heterogeneity in a paper are the variation in confidence intervals, the significance level (*p*-value), and the I2 value (Higgins et al., 2002; Lorenc et al., 2016).

When appraising systematic reviews and meta-analyses, one of the key challenges is assessing the level of heterogeneity present in the study. According to Cochrane's Handbook, systematic reviews and meta-analyses should only be considered when the included studies exhibit a certain degree of homogeneity. Combining studies that address different topics can lead to meaningless results. Analogy-wise, one should avoid combining apples and oranges unless discussing a broader topic such as fruit. If a systematic review aims to investigate various interventions for a specific condition, each intervention should be analyzed separately to ensure high-quality results (Higgins et al., 2002).

#### Quality and bias assessment

Assessing the quality and bias of included studies is a critical step in conducting systematic reviews. Various tools can be utilized based on the type of studies included. Examples of such tools include the Cochrane Risk of Bias 2.0 Tool for randomized controlled trials, the Newcastle Ottawa scale for non-randomized studies, and the CASP Appraisal Checklist and LEGEND Evidence Evaluation Tool for mixed methods (Step 6 Assess Quality of Included Studies—Systematic Reviews—LibGuides at University of North Carolina at Chapel Hill). It is important to note that not all studies are alike, and the assessment of quality and bias should be performed diligently to ensure the overall quality of the paper. Contacting the authors of studies that lack complete or missing data is necessary to ensure accurate results (Relevo and Balshem, 2011; Smith et al., 2011).

#### Search strategy

Developing a well-conducted search strategy is crucial for an optimal systematic review and meta-analysis. Multiple databases should be included in the search, as diagnostic search filters are not well-developed for these purposes. Commonly searched databases include Medline, EMBASE, Cochrane Central Register of Controlled Trials, CINAHL, and PsychINFO. It is important to utilize a comprehensive range of vocabulary and MESH terms to obtain the best results. However, challenges can arise in the search strategy, as errors can lead to poor-quality systematic reviews. A study evaluating 130 systematic reviews found that 92.7% had errors in their search strategies, with missing terms being the most common mistake (Salvador-Oliván JAco-Cuenca and Arquero-Avilés, 2019).

Choosing appropriate MESH terms is a critical step that directly impacts the quality of a systematic review and meta-analysis. It is essential to ensure the adequacy and suitability of the MESH terms used for the topic under review. Inadequate searching or failure to comprehensively search the relevant topic may stem from limited knowledge or the overwhelming availability of various components. While expanding the search and actively seeking studies to include in the systematic review and meta-analysis is desirable, it can increase the workload, as researchers will need to assess and filter a larger number of collected papers to identify relevant studies (Relevo, 2012).

## Conclusion

Conducting a comprehensive assessment of study quality in systematic reviews is vital for establishing the credibility and reliability of the evidence presented. This entails formulating clear research questions, implementing rigorous search strategies, employing strict inclusion criteria, utilizing appropriate evaluation tools, conducting a thorough evaluation of study quality, employing suitable data synthesis methods, disclosing conflicts of interest, acknowledging limitations, and addressing bias within the systematic review itself. By adhering to these guidelines, researchers can ensure the validity and usefulness of their systematic reviews, providing valuable insights for decision-making and guiding future research and practice.

## Author contributions

NS: Conceptualization, Methodology, Writing—original draft, Writing—review & editing. AS: Writing—original draft, Writing—review & editing. AlR: Writing—original draft, Writing—review & editing. MTH: Writing—original draft, Writing—review & editing. AbR: Writing—original draft, Writing—review & editing. II: Writing—original draft, Writing—review & editing. MEH: Writing—original draft, Writing—review & editing. MEA: Writing—original draft. OF: Writing—review & editing, Writing—original draft.
